# Quantifying semi-volatile organic contaminants in solution by internal standard addition method requires prompt addition of the internal standards

**DOI:** 10.1038/s41598-020-74688-4

**Published:** 2020-10-19

**Authors:** Ochan Otim, Jesus Rocha

**Affiliations:** Environmental Monitoring Division, City of Los Angeles, Playa Del Rey, CA 90293 USA

**Keywords:** Environmental sciences, Environmental chemistry, Environmental monitoring

## Abstract

The precision and accuracy of quantifying semi-volatile organic compounds (SVOCs) in solution by GC/MS, particularly when volume errors are unpredictable or difficult to control, are improved by utilizing internal standards (IS). Not obvious though is the extent to which timing IS addition affects measurement. To illustrate this fact, the mean concentrations of 60 SVOCs (40 or 80 μg/mL) in two identical solutions into which IS were added at different times are compared in this study. In one solution, IS were added promptly on preparation (*reference*); in the other, IS were added after 36 days of incubation (*treatment*). To investigate the role that temperature might play here as well, equal fractions of each solution were incubated at − 20 °C, 4 °C or 22 °C. Results, as determined by one-way ANOVA, show that there were no differences between the reference solutions at the beginning and after 36 days (*F*_3,236_ = 0.244, *p* = 0.865), but that significant differences exist between the reference solutions collectively and the treatment irrespective of temperature (*F*_6,413_ = 6.76, *p* = 1.99e^−06^). These results, confirmed by a post hoc analysis, suggest that uncertainty is introduced into SVOC quantitation when internal standards are not added promptly into SVOCs solutions on preparation.

## Introduction

Semi-volatile organic compounds (SVOCs) of anthropogenic origin are ubiquitous in the environment due to their widespread uses in industrial and domestic applications. Some of these SVOCs are hazardous and their presence, usually at trace-levels in indoor air, sediments, biological tissues and water, is carefully monitored^1–4^. To do so, appropriate environmental matrices are sampled and from it, SVOCs are purified in ways that preserves as much as possible both the nature and extent of contamination at the source. To increase certainty during this process, surrogate compounds are added to samples prior to extraction, particularly when uncontrolled sample losses are anticipated^5^. These surrogates are compounds with similar analytical characteristics to SVOCs but not present in samples. The reasoning underlying correcting the impact of losses during purification this way is that the ratio of SVOCs instrumental responses to those of surrogates would have *less* variability than the responses of the SVOCs alone.

To quantify the purified SVOCs by GC/MS, most environmental laboratories prefer to add a known and a constant amount of internal standards (IS) into the SVOCs solutions as well. Using IS as an aid this way also requires generating a calibration curve against which SVOC concentrations in environmental extracts are determined. In our hands, we have noticed that measuring SVOCs concentrations by this practice, at times, have led to larger values when IS were not added on the day of solutions preparation. Similar anomaly was pointed out in principle a while ago^6^ and because no study has so far been done to verify the existence of this phenomenon (with potentially a costly regulatory consequence), we chose to compare statistically the concentrations of SVOCs in two identical sets of solutions which were stored at three commonly used temperatures as described below.

## Material and methods

### Study design

**Figure 1:**
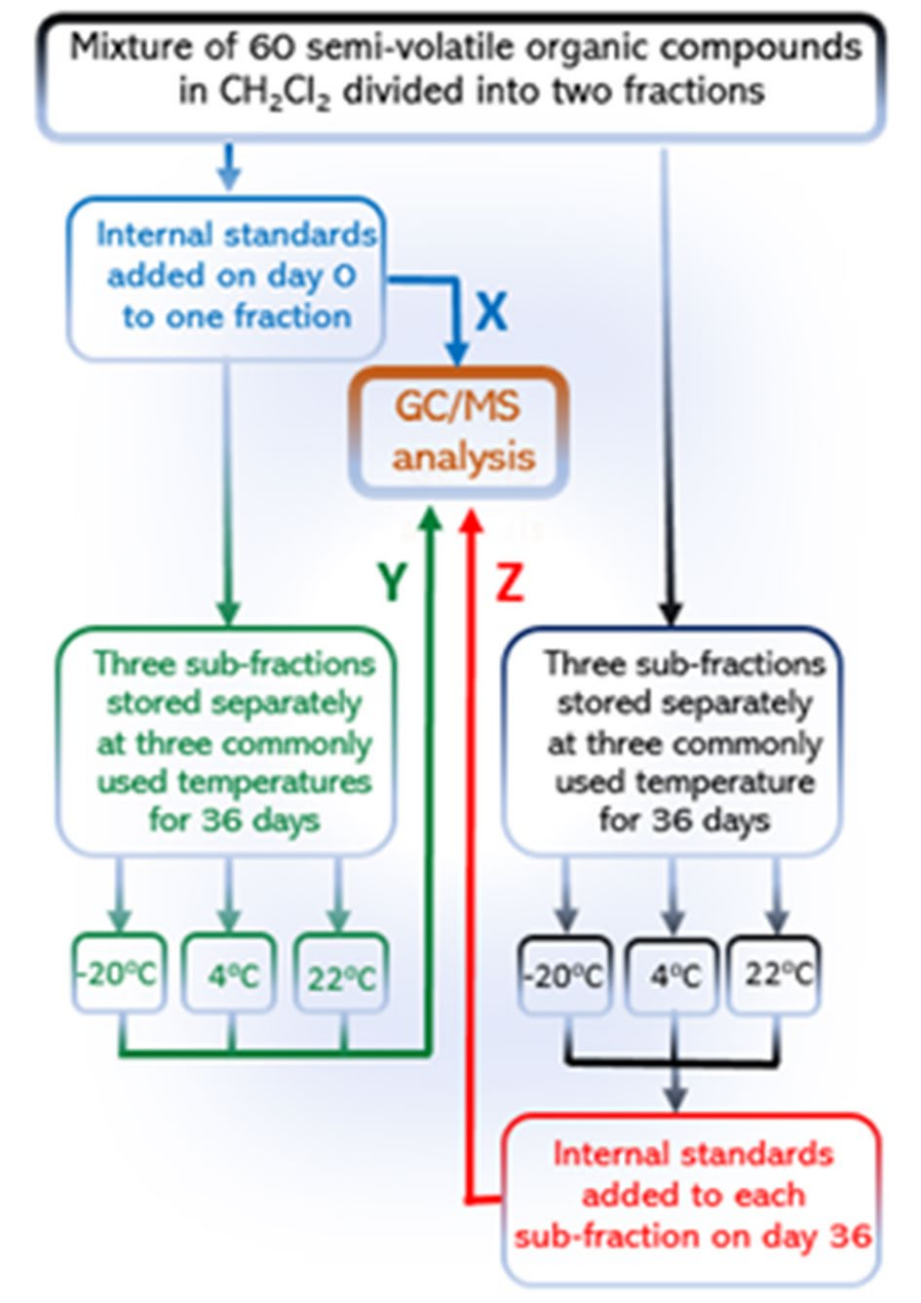
A schematic showing the design of this study. Step **X** provided the *reference* concentrations on day 0, the day of samples preparation; Step **Y** provided the *reference* concentrations after 36 days as measured by ‘aged’ internal standard (i.e., added on day 0); Step **Z**, the *treatment*, provided concentrations as measured by freshly added internal standards on day 36 after storage.

The schematic of this study is shown in Fig. 1. Briefly, a homogenous mixture of 60 SVOCs was split into two fractions and to one fraction (*reference*), a mixture of five deuterated internal standards (1,4-dichlorobenzene-d_4_, naphthalene-d_8_ and acenaphthene-d_10_, chrysene-d_12_ and perylene-d_12_)^7^ were added, each at 40 μg/mL. The five deuterated IS were selected so that the retention time (RT) of any of the 60 SVOCs was within 0.8–1.2 min of at least one internal standard. (Note that by requiring an SVOC signal be 0.8 to 1.2 min from an IS signal also assures the accuracy of GC retention times^7,8^) This first half was subdivided into three equal fractions and analyzed promptly by GC/MS to acquire triplicate reference data (Step **X**, coded blue in Fig. 1). The reference solutions were then stored separately in the dark at − 22 °C, 4 °C or 22 °C over a period of 36 days and analyzed again to determine if the aged IS/SVOCs solutions would reproduce the reference concentration values determined on the day of solution preparation (Step **Y**, coded green in Fig. 1). To the second fraction, the mixture was similarly subdivided into three equal fractions on day 0, and similarly stored but without IS until day 36.

All six sub-samples were analyzed on day 36 in a single batch. In the end, three sets of data were acquired. These data represent seven groups of timing the addition of IS: **X**—the reference samples with IS on day 0 at a single temperature (22 °C; color coded blue in all figures), **Y**—the aged reference samples with IS (color coded green in all figures), and **Z**—the treatment samples without IS until day 36 (coded red in figures). To assure precision, **X** measurements were in triplicate, **Y** and **Z** in duplicates.

### Sample preparation

All chemicals were obtained from Sigma-Aldrich (St. Luis, MO, USA). A mixture of 60 SVOCs was prepared to contain benzidine and 3,3′-dichlorobenzidine at 80 μg/mL and the rest at 40 μg/mL by mixing 240 μL each of EPA CLP Semivolatile Calibration Mix (1000 μg/mL, CRM 506508) and EPA 8270 Benzidine Mix (2000 μg/mL, CRM 48467) and topping up to 6000 μL with CH_2_Cl_2_. This solution was subdivided into six 1-mL colorless GC/MS vials (Thermo Scientific 11 mm Glass Crimp Top Vials), 1000 μL to a vial. To three of these vials, 20 μL of EPA 8270 Semivolatile Internal Standard Mix (2000 μg/mL, CRM 48902) was added, capped tightly and analyzed by GC/MS to acquire the baseline data (**X** in Fig. 1), then stored for 36 days before analysis one more time for changes in concentrations, if any (**Y**). The three remaining sample vials were also capped tightly (Restek 11 mm Aluminum Crimp Seals w/Septa) before storage without IS until day 36. These were the treatment samples (**Z**).

To test how temperature affect measured values, a vial with IS on day 0 (one of the three **X** vials) and one without IS (one of the three **Z** vials) were paired up and stored at − 20 °C, 4 °C or at 22 °C as described earlier^9^. Then on day 36, IS was added to **Z** and all six samples analyzed by GC/MS in a single batch as previously described^2,10^. The IS added in **Z** on day 36 came from one of four sealed Amber glass vials of the same lot used in X and Y on day 0. This was done to minimize experimental errors arising from possible differences in IS concentrations between lots, hence making measurements impossible to compare.

Prior to GC/MS analysis (below), samples were equilibrated at room temperature for 30 min and their volumes checked with a 0–1000 μL range pipette (Eppendorf Research Plus; Hamburg, Germany) with the intention of adjusting to 1 mL with CH_2_Cl_2_ if it were necessary. However, all volumes were found to be 1 mL; adjustment was therefore not necessary. The latter implies that evaporating was under control in all samples during the course of this study. For practical reasons, volume measurement, because of convenience and its widespread acceptance, was preferred here over weighing sample vials before and after incubation as a way of assessing the role evaporation plays in the concentration evaporation.

### GC/MS quantitation

A five-point calibration curve was generated for each compound of interest on the basis of the ratio of SVOC responses in 5, 10, 20, 40, 60 μg/mL CH_2_Cl_2_ solutions to that of a constant amount of semivolatile internal standard prepared above (final concentration: 40 μg/mL, benzidine and 3,3′-dichlorobenzidine at 80 μg/mL) in each calibration solution^7^. The calibration curve was then used to relate raw GC/MS measurements to the known concentrations of the 60 SVOCs in each calibration sample^7,10^. The GC/MS system was a dedicated HP 6890 GC /HP 5972 MSD mass detector system with a DB-5MS capillary column (30 m × 0.250 mm narrow-bore diameter × 0.25 μm film, Agilent). The MSD was capable of detecting masses in the range of 35–500 amu at 1 s/scan and was operated at 70 eV. The GC/MS column performance and the injection port inertness were verified by analyzing 1 µL a GC/MS tuning solution containing 50 ng/µL each of decafluorotriphenylphosphine (DFTPP), 4,4′-DDT, pentachlorophenol, and benzidine. Passing system suitability check meant the pentachlorophenol and benzidine tailing factors were less than 5 and 3, respectively, and the degradation of DDT to DDE and DDD was less than 20%. The column temperature program was as follows: 38 °C initially for 4 min followed by an 8 °C/min increase to 270 °C, and at 10 °C/min to 280 °C and holding for 11 min until the last compound (benzo[*ghi*]perylene) was detected. The injector was a splitless Grob-type maintained at 270 °C. Other conditions were: sample volume, 0.5 μL; helium carrier gas flow rate, 1 mL/min; MS transfer line temperature, 300 °C; MS source temperature was 230 °C. Samples were analyzed in duplicates to ensure precision. The coefficients of variance (ratios of standard deviation to mean) were ≤ 1% in all cases. The method detection limits (MDLs) for 56 of the SVOCs studied are provided in Table 2 of reference^9^.

On each day of analysis, a continuous calibration verification standard solution (CCV) was prepared by mixing 20 μL each of the following stock solutions (or a 1:1 dilution where necessary) from Supelco Analytical (Bellefonte, PA) and toping up to 1000 μL with dichloromethane: TCL Base-Neutrals Mix (1000 μg/mL), SS TCL Benzidines Mix (2000 μg/mL), SSTCL Phenols Mix 2000 μg/mL), 8270 Surrogate Standard (2000 μg/mL), TCL Hazardous Substances Mix 1000 μg/mL), and SS TCL Polynuclear Aromatic Hydrocarbons Mix (1000 μg/mL). The resulting mixture also contained the same mixture of 60 organic acids, bases and neutral compounds considered by the EPA as priority environmental pollutants (see Online Resources 1); 13 were calibration check compounds, and four were system performance check compounds. For quantitation, 20 μL of internal standards (described earlier) were added at the 40 μg/mL level to each 1000 μL sample vial.

### Statistical analyses

One-way analysis of variance (ANOVA) was used to compare the seven different groups of data for significant differences (set at *p* < 0.05). The working hypothesis was that the median concentrations of all groups were identical. The alternative hypothesis was that at least one median would differ from the others. Equal variance was assumed and confirmed by a modified Levene’s test after the fact.

Outliers were determined by computing the 1.5 interquartile concentration ranges (IQR) for each of the three experiments. Any concentration which fell statistically below or appears above IQR was then considered an outlier.

Standardized concentration values (mean = 0 and standard deviation = 1 for each SVOC) were explored using cluster analysis (CA) and principal component analysis (PCA) techniques to determine the diversity of SVOCs recoveries under conditions **Y** and **Z**, and to assess similarities between SVOCs recoveries within a group, if any.

## Results and discussion

In this study, we focused on a bulk property of solutions and as a result, we mostly used the mean concentrations of 60 SVOCs in our analysis. Parties interested in concentration changes and associated kinetics for each of the SVOC studied here are directed to a recently published reference^9^.

### Descriptive

**Figure 2:**
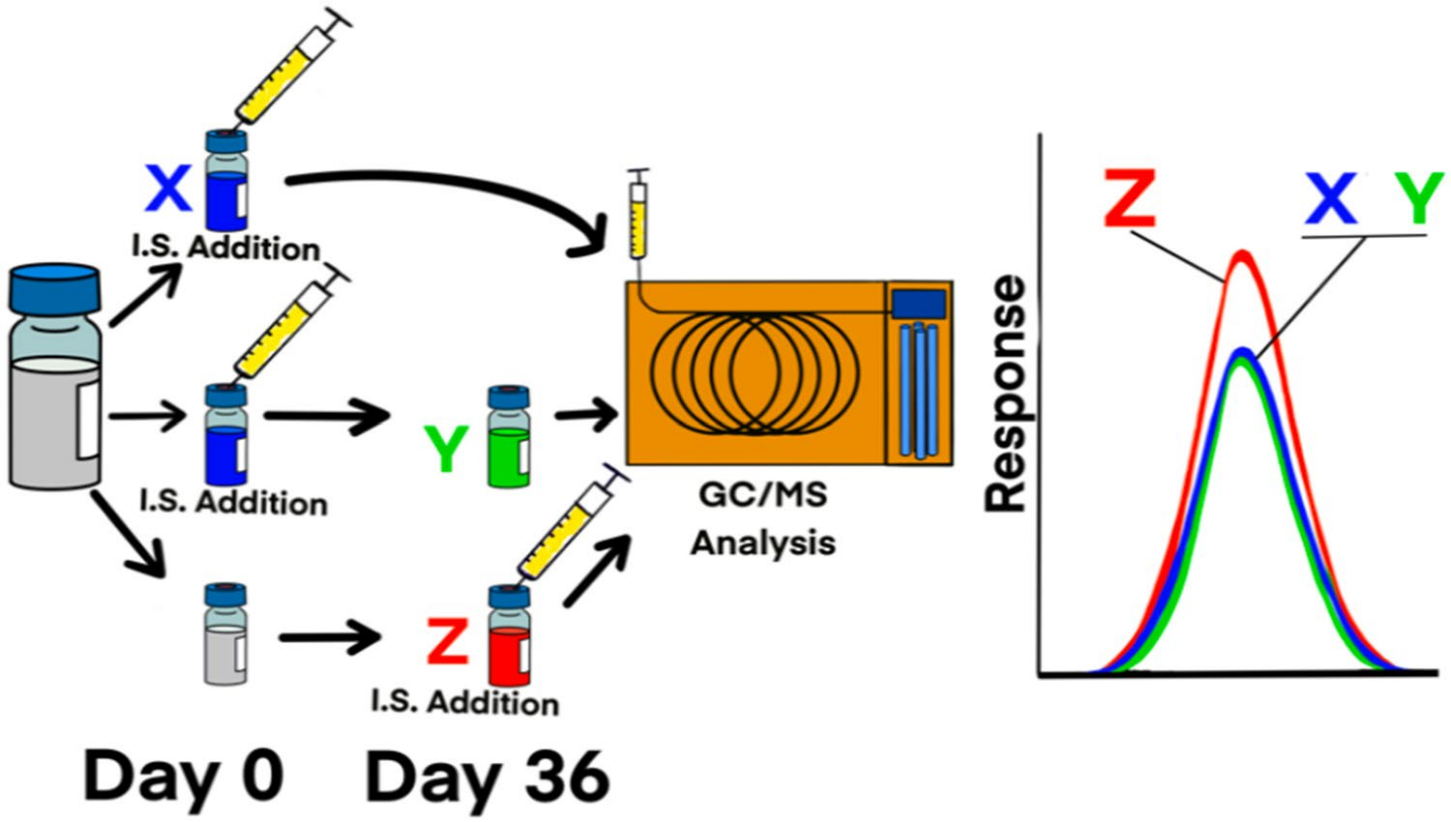
A schematic showing that steps **X** and **Y** (Fig. 1) led to identical results, but that step **Z**, led to higher SVOC concentration readings.

**Figure 3:**
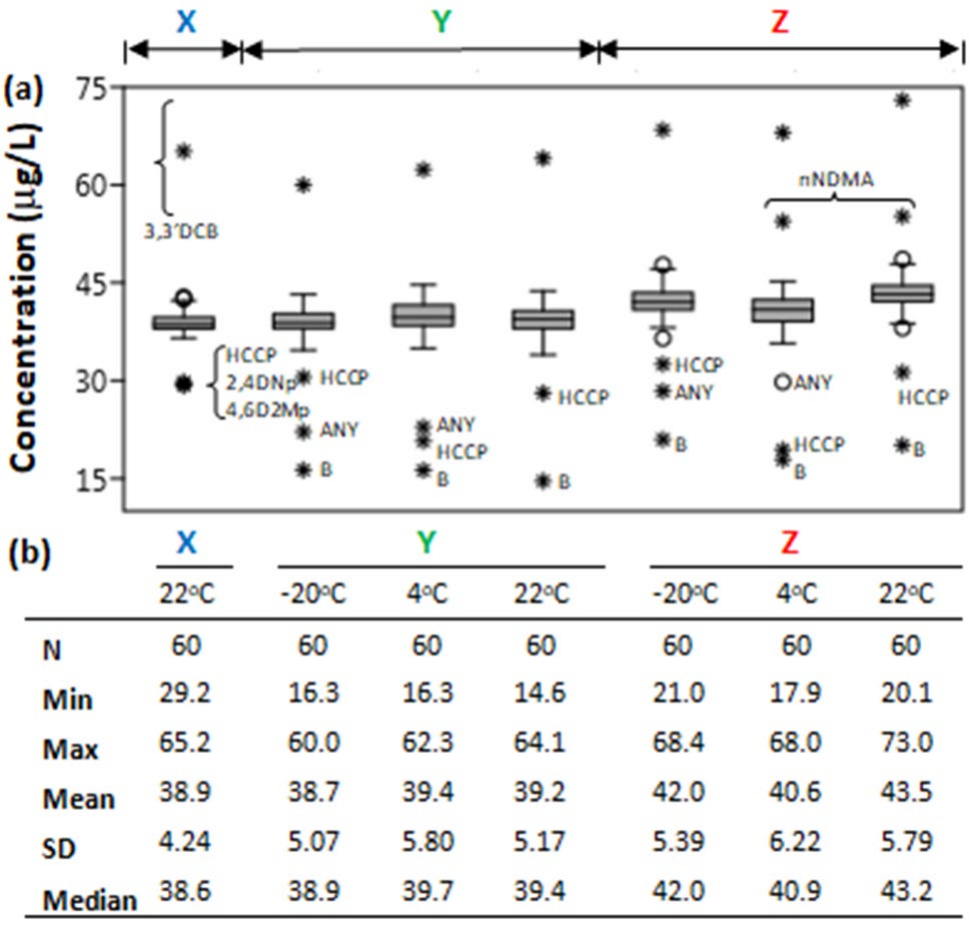
(**a**) Boxplots showing the distribution of the recoveries of the 60 SVOCs used in this study (Online Resources 1) as a function of temperature. Abbreviations have the following meanings: 3,3DCB: 3,3′-dichlorobenzidine; ANY: acenaphthylene; B: benzidine; HCCP: hexachlorocyclopentadiene; nNDMA: N-nitrosodimethylamine. (**b**) Summary statistics associated with the distribution are color coded as in Fig. 1 and aligned vertically below the corresponding boxplots. SVOCs were spiked at 40 μg/mL (benzidine and 3,3′-dichlorobenzidine at 80 μg/mL).

The results of the 420 concentration averages computed from 900 concentration values obtained in this study are presented visually in Fig. 2 and as boxplots in Fig. 3a. The numerical values together with the method detection limits of the GC/MS system used to obtain the averages (of duplicate measurements) are supplied in Online Resources 1. The summary statistics of the seven groupings of data arising from study design are presented in Fig. 3b.

These results show that the means and the medians of the distributions are similar, meaning the datasets have symmetrical distributions. They also show that the spread of data is limited to 4.2 μg/mL (37.9–42.1 μg/mL) in general when the most sensitive compounds indicated by abbreviations below the boxplots are excluded. Having said that, there is negligible difference between median reference concentration on day 0 (**X**, median: 38.6 μg/mL at 22 °C) and on day 36 [**Y**, medians: 38.9 μg/mL (− 20 °C), 39.7 μg/mL (4 °C) and 39.4 μg/mL (22 °C)]. However, there is a visible difference between the median concentrations acquired under the **X/Y** conditions on one hand and solutions aged without IS on the other hand (**Z**, medians: 42.0 μg/mL (− 20 °C), 40.9 μg/mL (4 °C) and 43.2 μg/mL (22 °C)). Comparatively, the median concentrations in the **Z** group are shifted to higher values by about 2 μg/mL on average. This shift appears to suggest that delaying addition of IS post-extraction could potentially lead to acquiring higher concentration values. To a casual observer, a 2 μg/mL concentration difference between samples with IS added on day 0 (**X** and **Y**) and those without (**Z**) may not raise as much concern. But to environmental regulators, this may amount to a violation of the law which may come with hefty fines.

It is tempting to attribute these observed differences to solvent evaporation. The futility of this attribution becomes clear when a scenario in which evaporation is eliminated is considered (i.e., sealed glass ampules of internal standards as used here on the two days of analysis). For example, for a fixed amount of IS added on day 0 to quantify *x* g of SVOC (*x*_0_ g) by an ideal GC/MS system, or much later on day *t* to quantify the same amount of SVOC (*x*_t_ g) by the same GC/MS system, no SVOC losses should be registered if indeed solvent evaporation is the major factor influencing measurements here (i.e., *x* = *x*_0_ = *x*_t_) since the final volume was made up to the volume on the day of preparation (i.e., 1 mL). This scenario is not supported by our data. Solvent evaporation, however, could make sense in the context of this study if evaporation of solvent in which IS was dissolved occurred before the internal standards were added on day 36. In which case, lower values instead would be obtained and not what is observed here.

Figure 3a, additionally, provides grounds for a few exceptions, however. The evidence presented therein inherently suggests that when quantifying benzidine (B) particularly in solution, stability during GC/MS analysis may as well be more important than the impact of timing IS addition. Spiked at the same 80 μg/mL level as it derivative 3,3′-dichlorobenzidine (3,3′-DCB), benzidine was the most affected compound in the mixture. Its concentration was not only ½ of 80 μg/mL in the reference sample (**X**) but fell to the lowest levels in all samples after 36 days. This is while the level of 3,3′-dichlorobenzidine remained fairly constant (slightly higher in Z, but nearly twice as high as initial benzidine) under all conditions. We believe that temperature at the GC/MS injection port is responsible for this huge loss of benzidine relative to 3,3′-dichlorobenzidine.

Note the temperature-dependence ordering of outliers (determined as described in the Material and methods section), from highest concentration value to lowest in Fig. 3a: − 20 °C, acenaphthylene (ANY) > hexachlorocyclopentadiene (HCCP) > benzidine (B); 4 °C, HCCP > ANY > B; 22 °C, HCCP > B. This pattern suggests the following: (1) that hexachlorocyclopentadiene measurement by GC/MS with the internal standards method will give a lower values, even on the day of solution preparation, (2) that relative to hexachlorocyclopentadiene and benzidine, measured acenaphthylene values could be lower in solutions stored at − 20 °C, but higher in solutions stored at 4 °C and 22 °C, and (3) that measurement of acenaphthylene is accurate and precise when it is stored at room temperature (i.e., the values are not outliers at 22 °C).

### Relative performance of internal standards

**Figure 4:**
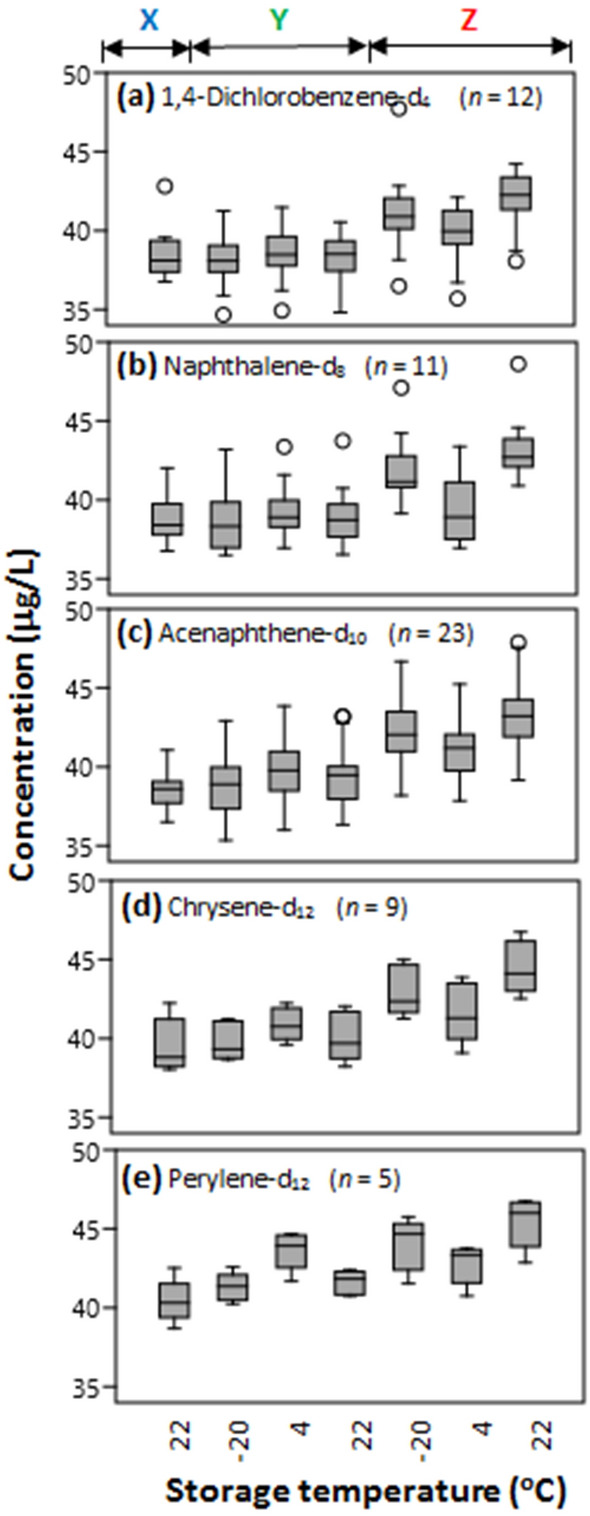
Boxplots of recoveries of 60 SVOCs in solution (each 40 μg/mL; benzidine and 3,3′-dichlorobenzidine at 80 μg/mL) as in Fig. 3 but broken down to values as measured by the respective internal standards (**a**–**e**) added on the day of preparation and analyzed by GC/MS immediately (**X**) and 36 days thereafter (**Y**), and when internal standards were added after 36 days of storage (**Z**). The number of SVOCs studied in each of the five cases (a-e) is given by the value of *n* in parenthesis. Conditions are color coded as in Fig. 1.

The 60 SVOCs studied required five IS to meet the need of having one every 0.8 to 1.2 min from an SVOC signal. The detailed groupings of SVOCs data around the five respective internal standards are presented in Fig. 4 by increasing retention times from Fig. 4a–e. Such IS-based display of distribution could be used to interrogate the relative performance of each internal standard, or the suitability of a GC/MS system to make precise measurement. For example, the systematic decrease in data spread from Fig. 4a–e could mean our condition of analysis was unsuitable for using 1,4-dichlorobenzene-d_4_, naphthalene-d_8_ and acenaphthene-d_10_ (Fig. 4a–c), or that the early eluting compounds are not stable and hence could not be measured precisely here.

In general, the median values for the early eluting compounds as measured by 1,4-dichlorobenzene-d_4_, naphthalene-d_8_ and acenaphthene-d_10_ (Fig. 4a–c) are similar for the **X** and the **Y** groups but collectively different when compared to the **Z** groups. Furthermore, the whiskers are longer by these three IS but very short-to-none in measurements based on chrysene-d_12_ and perylene-d_12_ (Fig. 4d–e). It can also be seen that the middle 50% of the distributions overlaps only within the **X** and the **Y** groups, signifying that the median values of **X** and **Y** on the one hand are separated by a large concentration from those of the **Z** groups.

### ANOVA

To test statistically whether timing the addition of IS to SVOCs solution introduces uncertainty in measurements as suggested above, the means of SVOCs concentrations in solutions spiked with IS on day 0 (reference: groups **X** and **Y**) were compared with those of identical solutions aged first for 36 days before spiking with IS (treatment: **Z** in Fig. 1). The results show that, whereas there were no difference between **X** and **Y** days (*F*_3,236_ = 0.244, *p* = 0.865), there was a statistically significant difference between groups **X** and **Y** on the one hand, and **Z** on the other hand as determined by one-way ANOVA (*F*_6,413_ = 6.763, *p* = 1.992e^−06^). This observation means the effect of delayed IS addition is significant. The *p*-value of 1.992e^−06^ indicates that H_*o*_ should be rejected because the seven-group means are not equal. The assumption of equal variances here was determined to be valid (*F*_6,413_ = 0.493, *p* = 0.814).

To determine which pairwise means differences were significant, a post hoc test (Tukey’s method) was conducted and the results revealed that six pairs were indeed significantly different (Table 1, *p* < 0 0.05), all of which invloves either **X** or **Y** on one side and **Z** on the other side. Four of the pairs involve pairing solutions stored without IS at room temperature (**Z** 22 °C) with the reference solutions on day 0 (**X** 22 °C) or on day 36 after storage at 4 °C (**Y** 4 °C) or 22 °C (**Y** 22 °C). Two additional pairing involves solution without IS after 36 days at − 20 °C with the reference solution (1) on day 0 (**X** 22 °C), or (2) after a − 20 °C storage over 36 days (**Y** − 20 °C).

**Table 1:** Results of a post hoc test conducted to determine exact pairs of experimental conditons with significant mean difference. Tukey's Q below the diagonal, *p*(same) above the diagonal.

	**X** 22 °C	**Y** − 20 °C	**Y** 4 °C	**Y** 22 °C	**Z** − 20 °C	**Z** 4 °C	**Z** 22 °C
**X** 22 °C		1.00	1.00	1.00	0.03^a^	0.58	8.46e^−05a^
**Y** − 20 °C	0.255		0.99	1.00	0.01^a^	0.46	3.66e^−05a^
**Y** 4 °C	0.796	1.051		1.00	0.12	0.90	1.00e^−03a^
**Y** 22 °C	0.436	0.690	0.361		0.06	0.78	3.00e^−04a^
**Z** − 20 °C	4.504	4.759	3.708	4.069		0.78	0.76
**Z** 4 °C	2.472	2.727	1.676	2.037	2.032		0.06
**Z** 22 °C	6.599	6.853	5.802	6.163	2.094	4.126	

**Z** is the reference samples; **Y** is the reference samples analyzed again after 36 days of incubation at different temperature. **Z** samples are **Y** with no internal standards until day 36.

^a^Significant difference.

### Cluster analysis

**Figure 5:**
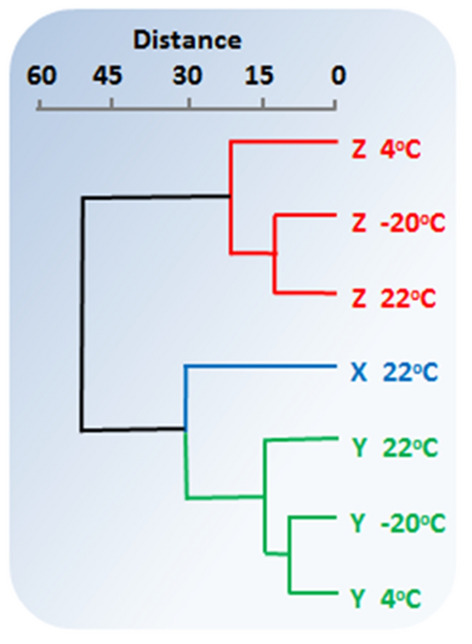
Cluster analysis results showing similarity between groups **X** and **Y**, and dissimilarity between **X** or **Y** and **Z** at all three temperatures of this study. Conditions are color coded as in Fig. 1. (Algorithm: Ward's method; cophenetic correlation: coefficient: 0.64).

The ANOVA pattern is confirmed by CA results as shown in Fig. 5. The CA results are based on a 7 × 60 mean concentration data matrix derived from the seven conditions listed in Table 1 and the concentrations of the 60 SVOCs collected in this study. Clearly, there are two distinct groupings in the data at a similarity distance of 30 with the entire **Z** samples group together to form a single cluster. In that cluster, the samples stored without IS at room temperature (22 °C) and in the freezer (− 20 °C) are more alike than with samples store in the refrigerator (4 °C). We offer no explanation for this association between these two extreme temperatures at this time.

### Inference

To contextualized the general meaning of our findings here, the following considerations may be helpful: (1) that the physical components of each of the seven groupings (Fig. 5) were the same (60 SVOCs), (2) that concentrations were identically measured, and (3) that loss of SVOCs or IS through mechanisms such as adsorption to container walls or breakdown with time was unavoidable. Under those considerations, adding IS to SVOCs solutions after 36 days but just before GC/MS analysis—which would mean not allowing IS to age alongside the SVOCs—should introduce concentration variability as a consequence. That is, the longer the addition of internal standard is delayed, the higher will be variability in measurement.

**Figure 6:**
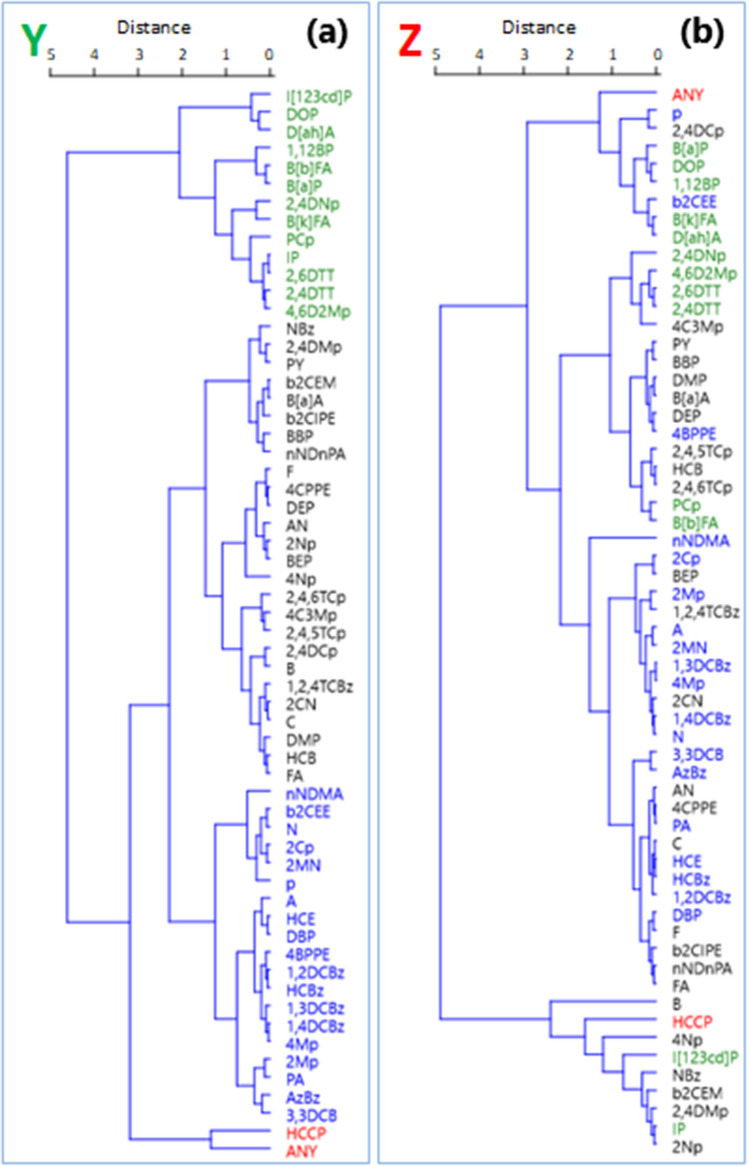
Comparing cluster analysis results of standardized recoveries of 60 SVOCs as measured (**a**) when internal standards were added on day 0 of samples preparation and aged for 36 days (condition **Y**) and (**b**) when internal standards addition was delayed until after 36 days of incubation (condition **Z**). Abbreviations represent compounds; the identities are supplied in Online Resources 1). Four groupings were identified in **Y**; members of a grouping are color-coded identically to facilitate tracing in **Z**. (Algorithm: Ward's method; cophenetic correlation: coefficients: 0.81).

That being said, grouping results by temperature as presented above may be obscuring other useful observations that may lead to identifying more potential causes of differences between conditions **Y** and **Z**. Indeed, the apparent ‘differences’ in SVOC concentrations observed here may as well be due to additional mechanisms at the molecular level which are yet to be defined. For example, evaluating the **Y** and **Z** data at molecular level by exploratory cluster analysis in a multivariate space suggests that there is an order in which the 60 SVOCs are quantified by the five (5) internal standards used here, and that the order is based mostly on RT of the SVOCs listed in Online Resources 1. The results of this cluster analysis (displayed in Fig. 6) show the existence of four different groupings in the data, the basis of which is largely RT under the reference conditions **Y** (except for HCCP, hexachlorocyclopentadiene and ANY, acenaphthylene color coded red in Fig. 6a). These groupings by RT are lost under the treatment conditions **Z** when addition of internal standards is delayed until day 36 (Fig. 6b). Note also the parallel between the orderly groupings displayed for **Y** in Fig. 6a and the pattern of data spread common to both **Y** and **Z** in Fig. 4. It means the parallel is not enough to conclude that precision is the basis of the groupings in Fig. 6a; otherwise Fig. 6b would also display the same orderly groupings. We offer not further explanation here as well.

**Figure 7:**
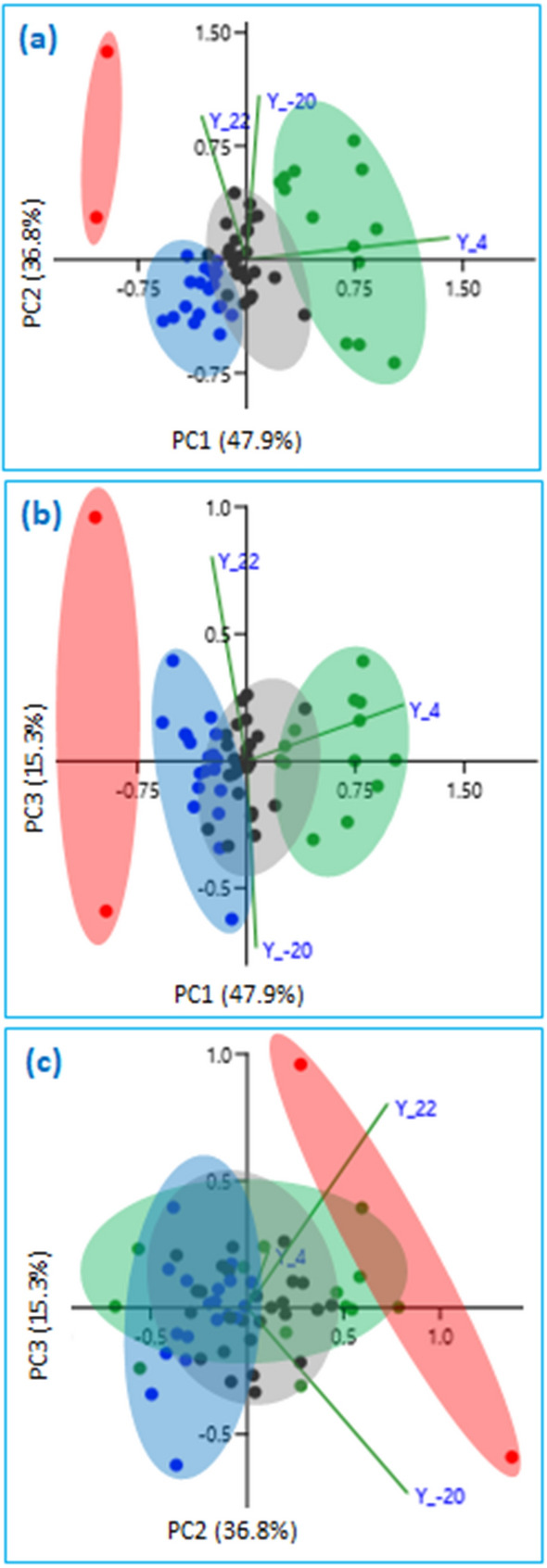
SVOCs groupings by principal components analysis resulting from concentration variance under conditions **Y**. Vectors include recoveries at − 22 °C (Y_ −  20), 4 °C (Y_4) and 22 °C (Y_22). The first three PCA axes are shown; each plot displays a different combination of axes: (**a**) axes 1 and 2, (**b**) axes 1 and 3, (**c**) axes 2 and 3. Approximate area coverage of the four groupings identified by CA (Fig. 6) are similarly color-coded.

To verify perhaps at the molecular level the groupings identified in Fig. 6a, PCA was also performed using the same standardized data. The results, shown in Fig. 7, confirms the orderly alignment of CA groupings by retention times in Fig. 6a (i.e., green → black → blue → special case grouping red as mentioned earlier, condition **Y**). The cumulative variance for all three axes (PC1, PC2 and PC3) explained nearly 100% of the variation of the standardized concentration data under conditions **Y**. The first axis explained a dominant gradient between SVOCs recoveries on the basis of RT, with positive loadings associated with SVOCs with the highest RT values and negative loadings associated with SVOCs with lower RT values. Along this axis, the mostly early eluting SVOCs (blue) loaded negatively along PC1 while the mostly late eluting SVOCs (green) loaded positively (Fig. 7a,b). Vectors Y_4 (condition Y at 4 °C) and Y_− 20 (condition Y at − 20 °C) have positive values, but with the former having a much larger value suggesting that perhaps storing at 4 °C a mixture of IS and the late eluting SVOCs, which are mostly polycyclic aromatic compounds, should be preferred over − 20 °C. The SVOCs grouping color coded black is not explained by the variables we measured (concentration and temperature). The second axis describes internal gradient within each group (except red), the basis of which is not clear at this time. All the three vectors Y_22 (condition Y at 22 °C), Y_4 and Y_− 20 are positive along this axis. The third axis (Fig. 7c) explains only 15.3% of the variation among conditions Y, but explains the difference between lability represented by one of the two red group members (HCCP) loading strongly and negatively, and the unusually stable group member (ANY) loading strongly and positively along this axis.

A similar application of PCA, a statistical method based on principle different from those underlying CA, on the **Z** data has no discernible groupings (Online Resource 2).

### Roadmap to follow-up studies

The limited goal of this study—which was to determine statistically whether timing addition of internal standards had any impact on reported measurements under conditions mimicking accepted laboratory practices in a typical environmental laboratory—was met. In the process of meeting the goal, opportunities towards addressing the concentration differences laid bare by results of this study emerged. These opportunities include understanding (1) the underlying complexity of environmental extracts such as those encountered here, (2) the possible chemical reactions between internal standards and SVOC components, or among SVOC components themselves, (3) the adsorption of SVOCs to container walls and (4) the need, or lack thereof, for gravimetric determination of mass as opposed to volumetric measurements for quantifying changes.

Reducing the complexity of the sample mixture could lessen any inter-compound interactions, if any. To do so would mean (1) quantify the internal standards using an inert reference materials such as deuterated alkanes, (2) studying the 60 SVOCs targeted here individually, and (3) comparing changes in the concentration of identical mixtures (or individual pairs) of deuterated/undeuterated forms of the same internal standards outside the mixture to ensure that they are actually stable under all the storage conditions common in environmental laboratories. The latter is particular relevant considering that results here show a significant difference in the scores for the **Z** setup (mean = 41.0 ug/mL, standard deviation = 1.0 ug/mL) when compared to the scores of the **Y** setup (mean = 38.2 ug/mL, standard deviation = 0.9 ug/mL); *t*(6) = 4.334, *p* = 0.005 (for 1,4-dichlorobenzene, naphthalene, acenaphthene and chrysene concentrations measured using their four deuterated internal standard analogs 1,4-dichlorobenzene-d_4_, naphthalene-d_8_, acenaphthene-d_10_ and chrysene-d_12_, respectively). Even puzzling, the benzidine level dropped markedly from 38 μg/mL (**X**) to 20 μg/mL after 36 days and yet the **Z** mixtures still gave higher results, almost irrespective of the temperature (Online Resources 1 and Fig. 3a).

Weighing the samples vessel at start and at the end of the 36 days would have detected granular variations in volume (although we believe solvent evaporation was not a problem since volumes were checked using analytical pipette on the day of samples preparation and at the end after 36 days). Alternatively, storing all study samples in sealed vials (and not only internal standards as was done here) during the temperature trials could have removed evaporation as a factor in this study. The latter is not to suggest that this course of action be adopted for routine analysis of environmental extracts because it is impractical and unnecessary.

Lastly, there is need to investigate systematically the inertness of, and leaks from, crimped septa of commonly used vials in environmental laboratories. Publicly available literature appears to lack adequate information to this effect. In the meantime, adding internal standards to SVOCs extracts on the day of sample preparation, or soon thereafter, is advised in order to avoid concentration ambiguity.

## Conclusion

We have shown that quantifying 60 SVOCs by adding internal standards much later into SVOC solutions can lead to a difference in measured values. Whereas no concentration changes were observed after incubating SVOCs solutions containing internal standards for 36 days, significant differences were observed between concentrations of the aforementioned solutions and those in which internal standards were added much later on day 36. This variation with time, especially when an extended delay in analysis is anticipated, could be minimized by incubating internal standards and SVOCs together in solution. Further studies are needed to fully understand the mechanism of the variability observed here. An initial study hypothesis could be that the observed differences are a consequence of a set of factors affecting both the SVOCs and internal standards similarly, and irrespective of time. Some of these factors are suggested in the text above.

## Supplementary information

Supplementary Information.

## Data Availability

All data are available in the text and in Electronic Supplementary Material ESM_1.
