# Supplementary material for: Quantifying semi-volatile organic contaminants in solution by internal standard addition method requires prompt addition of the internal standards
Source: Sci Rep. 2020 Oct 19;10:17662. doi: 10.1038/s41598-020-74688-4 (PMC7572397; doi:10.1038/s41598-020-74688-4)

Quantifying semi-volatile organic contaminants in solution by internal standard addition method requires prompt addition of the internal standards

Ochan Otim*, and Jesus Rocha Jr.
Environmental Monitoring Division, City of Los Angeles, Playa Del Rey, CA. 90293

*** Corresponding Author**
E-mail address: ochan.otim@lacity.org (Ochan Otim). ORCID: 0000-0001-7272-4356
Tel: +1 (310) 648-5835; Fax: +1 (310) 648-5828

Electronic Supplemental Material Table ESM_1. Method detection limit, the mean and the standard deviation for each SVOC studied. Keys to the table are supplied at the bottom of page 3.

					X		Y						Z					
					22°C		-20°C		4°C		22°C		-20°C		4°C		22°C	
**Compounds**		**RT**	**SD**	**MDL**	**mean**	**SD**	**mean**	**SD**	**mean**	**SD**	**mean**	**SD**	**mean**	**SD**	**mean**	**SD**	**mean**	**SD**
**IS 1,4-Dichlorobenzene-d_4 for:**																		
N-Nitrosodimethylamine	**nNDMA**	6.78	0.02	0.56	42.82	0.71	41.25	0.90	41.47	0.13	37.26	4.82	47.73	7.31	54.41	2.58	55.18	2.76
bis(2-Chloroethyl)ether	**b2CEE**	11.33	0.01	0.18	36.77	0.11	34.68	0.28	34.94	0.21	34.82	0.11	36.50	0.62	35.71	0.71	38.07	0.30
Phenol	**p**	11.14	0.01	0.54	37.38	0.24	35.88	0.14	36.21	0.11	35.70	0.34	38.15	0.52	36.71	0.49	38.73	0.25
2-Chlorophenol	**2Cp**	11.38	0.00	0.63	39.46	0.24	38.55	0.28	39.10	0.05	38.76	0.16	41.32	0.23	40.00	0.59	42.76	0.18
1,3-Dichlorobenzene	**1,3DCBz**	11.71	0.00	0.14	38.49	0.22	38.14	0.21	38.43	0.16	38.53	0.04	40.84	0.07	39.82	0.25	42.22	0.02
1,4-Dichlorobenzene	**1,4DCBz**	11.88	0.00	0.17	38.27	0.19	37.99	0.05	38.54	0.14	38.53	0.01	40.97	0.08	39.91	0.11	42.32	0.25
1,2-Dichlorobenzene	**1,2DCBz**	12.18	0.00	0.17	39.08	0.14	39.24	0.18	39.68	0.21	39.67	0.09	42.31	0.13	41.41	0.01	43.59	0.24
bis(2-chloroisopropyl) ether	**b2ClPE**	12.50	0.01	0.20	36.90	0.16	38.06	0.03	38.41	0.74	38.65	0.11	40.24	0.56	40.00	0.05	41.46	0.28
2-Methylphenol	**2Mp**	12.43	0.01	0.24	37.95	0.29	37.34	0.35	37.71	0.03	38.07	0.17	40.48	0.18	39.15	0.35	41.84	0.26
Hexachloroethane	**HCE**	12.89	0.00	0.14	39.57	0.54	39.59	0.50	40.30	0.37	40.54	0.06	42.85	0.09	42.12	0.18	44.23	0.37
N-Nitroso-di-n-propylamine	**nNDnPA**	12.76	0.02	0.21	37.42	0.40	38.53	0.18	39.46	0.48	39.53	0.04	41.14	0.37	40.89	0.04	42.80	0.98
4-Methylphenol	**4Mp**	12.77	0.02	0.30	37.92	0.25	37.51	0.14	37.96	0.23	37.98	0.28	40.06	0.63	39.14	0.61	41.29	0.45
**IS Naphthalene-d_8 for:**																		
Nitrobenzene	**NBz**	13.05	0.01	0.18	38.09	0.43	38.12	0.47	38.28	0.21	37.91	0.11	40.89	0.01	36.95	3.32	42.18	0.29
Isophorone	**IP**	13.58	0.02	0.22	36.77	0.19	38.33	0.30	38.89	0.11	38.49	0.08	40.81	0.21	37.47	3.55	42.10	0.54

					X		Y						Z					
					22°C		-20°C		4°C		22°C		-20°C		4°C		22°C	
**Compounds**		**RT**	**SD**	**MDL**	**mean**	**SD**	**mean**	**SD**	**mean**	**SD**	**mean**	**SD**	**mean**	**SD**	**mean**	**SD**	**mean**	**SD**
**IS Naphthalene-d_8 for *cont.* :**																		
2-Nitrophenol	**2Np**	13.71	0.01	0.44	39.36	0.62	36.98	0.18	38.37	0.46	37.67	0.15	41.14	0.38	37.52	3.27	42.57	0.91
2,4-Dimethylphenol	**2,4DMp**	13.86	0.01	0.57	42.01	0.30	43.20	0.41	43.37	0.09	43.74	0.30	47.11	0.57	42.43	3.32	48.61	0.06
bis(2-Chloroethoxy)methane	**b2CEM**	14.06	0.01	0.21	38.41	0.25	38.31	0.31	38.87	0.03	38.72	0.05	41.08	0.18	38.08	3.17	42.71	0.25
2,4-Dichlorophenol	**2,4DCp**	14.21	0.01	0.46	39.76	0.25	39.87	0.52	39.74	0.71	39.73	0.06	42.79	0.13	40.43	2.13	43.86	0.04
1,2,4-Trichlorobenzene	**1,2,4TCBz**	14.37	0.01	0.18	38.74	0.34	39.52	0.13	39.99	0.13	39.63	0.14	42.75	0.24	41.12	0.11	43.82	0.24
Naphthalene	**N**	14.52	0.01	0.23	37.97	0.28	36.97	0.03	37.37	0.16	37.28	0.06	39.86	0.29	38.92	0.13	41.01	0.19
Hexachlorobutadiene	**HCB**	14.81	0.00	0.14	37.80	0.54	38.83	0.20	39.28	0.08	39.18	0.05	42.46	0.53	40.88	0.23	43.22	0.23
4-Chloro-3-methylphenol	**4C3Mp**	15.67	0.01	0.57	40.49	0.58	40.82	0.32	41.58	0.31	40.74	0.13	44.22	1.04	43.39	0.11	44.58	1.22
2-Methylnaphthalene	**2MN**	15.93	0.01	0.21	37.22	0.24	36.51	0.30	36.96	0.02	36.54	0.04	39.14	0.45	38.46	0.04	40.89	0.49
**IS Acenaphthene-d_{10} for:**																		
Hexachlorocyclopentadiene	**HCCP**	16.27	0.00	0.05	29.72	2.62	30.53	0.83	20.73	0.61	28.12	0.98	32.53	0.89	19.39	0.09	31.27	1.39
2,4,6-Trichlorophenol	**2,4,6TCp**	16.53	0.02	0.39	39.25	0.44	39.98	0.45	40.98	0.43	39.86	0.32	43.50	0.92	41.93	0.11	44.16	0.52
2,4,5-Trichlorophenol	**2,4,5TCp**	16.57	0.01	0.17	38.57	0.23	39.29	0.28	39.78	0.21	38.94	0.34	42.61	1.03	40.82	0.07	43.13	0.86
2-Chloronaphthalene	**2CN**	16.91	0.01	0.24	38.13	0.22	38.67	0.15	39.23	0.30	38.99	0.20	41.63	0.32	40.65	0.05	43.19	0.13
Acenaphthylene	**ANY**	17.73	0.01	0.13	36.50	0.40	22.15	1.91	22.88	1.24	33.95	1.20	28.39	0.21	29.79	0.74	39.16	4.21
Dimethyl phthalate	**DMP**	17.56	0.02	0.13	38.59	0.20	39.51	0.23	40.32	0.10	40.04	0.14	42.87	0.42	41.58	0.08	43.89	0.81
2,6-Dinitrotoluene	**2,6DTT**	17.66	0.02	0.10	38.56	0.45	42.85	0.27	43.77	0.64	43.20	0.47	46.66	0.70	45.16	0.15	47.88	1.05
Acenaphthene	**AN**	18.10	0.01	0.39	37.70	0.38	37.35	0.16	38.20	0.06	37.96	0.08	40.39	0.21	39.75	0.06	41.90	0.22
2,4-Dinitrophenol	**2,4DNp**	18.18	0.01	0.67	29.69	2.81	39.75	0.08	42.87	2.06	39.47	1.10	45.16	1.39	42.06	0.26	44.65	2.85
2,4-Dinitrotoluene	**2,4DTT**	18.45	0.02	0.07	38.65	0.63	42.63	0.46	43.85	0.56	43.18	0.04	46.67	0.29	45.23	0.19	47.57	0.96
4-Nitrophenol	**4Np**	18.34	0.02	0.35	37.69	1.73	36.60	0.40	38.49	0.36	37.27	0.85	40.97	1.23	37.85	1.48	40.09	1.97
Fluorene	**F**	19.14	0.01	0.10	38.16	0.49	38.43	0.05	39.56	0.01	39.42	0.01	41.58	0.22	41.07	0.01	42.92	0.46
4-Chlorophenyl-phenyl ether	**4CPPE**	19.18	0.01	0.12	38.73	0.31	38.90	0.07	39.80	0.03	39.64	0.04	41.74	0.19	41.20	0.12	43.21	0.38
Diethyl phthalate	**DEP**	19.00	0.01	0.25	38.69	0.44	38.89	0.18	40.07	0.47	39.74	0.12	42.51	0.51	41.47	0.04	43.49	0.73
4,6-Dinitro-2-methylphenol	**4,6D2Mp**	19.27	0.02	0.44	29.20	2.24	35.35	0.11	37.18	0.52	36.34	0.11	40.21	0.63	38.96	0.23	41.61	1.12
Azobenzene	**AzBz**	19.49	0.01	0.32	37.73	0.60	35.56	0.01	36.00	0.33	36.54	0.26	38.19	0.32	38.05	0.08	40.12	0.25
4-Bromophenyl-phenyl ether	**4BPPE**	20.16	0.01	0.13	39.71	0.80	40.23	0.05	40.65	0.01	40.71	0.21	43.17	0.34	42.40	0.13	44.26	0.13
Hexachlorobenzene	**HCBz**	20.24	0.01	0.17	38.86	0.56	39.11	0.29	39.39	0.11	39.54	0.10	42.03	0.29	41.29	0.05	43.42	0.22
Pentachlorophenol	**PCp**	20.65	0.01	0.37	40.10	1.07	42.92	0.32	43.81	0.84	42.11	0.16	46.20	0.69	44.07	0.36	46.56	0.25

					X		Y						Z					
					22°C		-20°C		4°C		22°C		-20°C		4°C		22°C	
**Compounds**		**RT**	**SD**	**MDL**	**mean**	**SD**	**mean**	**SD**	**mean**	**SD**	**mean**	**SD**	**mean**	**SD**	**mean**	**SD**	**mean**	**SD**
**IS Acenaphthene-d_{10} for *cont.* :**																		
Phenanthrene	**PA**	21.07	0.01	0.1	39.1	0.3	38.1	0.2	38.6	0.3	38.9	0.1	41.0	0.1	40.6	0.2	42.4	0.2
Anthracene	**A**	21.17	0.01	0.1	38.6	0.3	38.6	0.0	38.8	0.1	39.4	0.3	41.2	0.6	40.3	0.1	42.9	0.2
Di-n-butyl phthalate	**DBP**	22.33	0.01	0.3	39.1	0.5	39.2	0.1	39.9	0.2	40.0	0.3	42.2	0.5	41.8	0.2	43.7	0.2
Fluoranthene	**FA**	23.46	0.01	0.3	41.1	0.7	42.3	0.2	42.9	0.3	42.8	0.1	45.1	0.0	44.8	0.0	47.0	0.5
**IS Chrysene-d_{12} for:**																		
Pyrene	**PY**	23.91	0.01	0.2	38.3	1.1	40.9	0.4	41.4	0.1	41.4	0.1	44.3	0.2	43.1	0.0	45.6	0.3
Butylbenzyl phthalate	**BBP**	25.35	0.01	0.2	38.8	1.0	41.2	0.2	42.2	0.3	42.0	0.3	45.0	0.0	43.9	0.1	46.7	0.7
Benzo[a]anthracene	**B[a]A**	26.36	0.01	0.2	38.0	0.4	39.8	0.2	40.4	0.1	40.2	0.2	43.2	0.6	41.9	0.2	44.4	0.5
Chrysene	**C**	26.44	0.02	0.2	38.4	0.3	38.9	0.2	39.6	0.3	39.2	0.1	42.0	0.3	41.3	0.3	43.6	0.2
Bis(2-ethylhexyl) phthalate	**BEP**	26.61	0.01	0.3	40.1	1.2	39.3	0.0	40.3	0.3	39.7	0.3	42.4	0.4	41.1	0.1	44.1	0.9
Di-n-octyl phthalate	**DOP**	28.34	0.00	0.2	40.2	0.4	38.8	0.0	41.6	0.1	39.5	0.0	42.2	0.1	40.9	0.1	43.5	0.5
Indeno(1,2,3-cd)pyrene	**I[123cd]P**	35.61	0.01	0.2	42.3	1.7	38.7	0.0	40.8	0.6	38.2	0.1	41.2	0.7	39.1	0.1	42.5	0.0
Benzidine	**B**	23.73	0.00	0.3	38.2	6.7	16.3	1.5	16.2	0.8	14.6	0.1	21.0	9.3	17.9	0.0	20.1	3.8
3,3'-Dichlorobenzidine	**3,3DCB**	26.31	0.00	0.5	65.2	4.2	60.0	2.9	62.3	0.1	64.1	0.4	68.4	1.4	68.0	0.1	73.0	2.6
**IS Perylene-d_{12} for:**																		
Benzo(b)fluoranthene	**B[b]FA**	29.22	0.00	0.2	40.1	0.2	41.6	0.1	44.5	0.2	42.4	0.7	45.7	1.1	43.8	0.3	46.6	1.8
Benzo(k)fluoranthene	**B[k]FA**	29.33	0.01	0.2	38.7	0.4	40.2	0.6	41.7	0.6	40.9	1.0	41.5	0.5	40.8	0.4	42.9	1.9
Benzo(a)pyrene	**B[a]P**	30.34	0.01	0.4	40.3	0.5	41.4	0.2	43.9	0.0	41.8	0.1	44.7	0.4	43.3	0.1	46.0	0.1
Dibenz(a,h)anthracene	**D[ah]A**	35.87	0.01	0.3	42.5	1.8	40.7	0.4	43.4	0.3	40.8	0.1	43.2	0.5	42.4	0.4	44.9	0.2
Benzo(g,h,i)perylene	**1,12BP**	37.18	0.01	0.1	40.6	1.7	42.6	0.2	44.7	0.0	42.2	0.2	44.9	0.2	43.6	0.2	46.7	0.3

KEY

MDL: Method detection limits (μg/L).

mean: Duplicate analysis (μg/L).

SD: Standard deviation (μg/L).

IS: Internal Standard.

RT: Mean of four (4) retention times (min) ± standard deviation **SD**.

X: Internal standard added to samples on day 0 and analysed immediately for reference.

Y: Same as **X,** but samples analysed after 36 days.

Z: Internal standard added to samples on day 36 and analysed on that day.

Electronic Supplemental Material ESM_2. SVOCs groupings by principal components analysis from clustering and variance among concentration measured under conditions Z. Vectors include recoveries at -22°C (Z_-20), 4°C (Z_4) and 22°C (Z_22). The first three PCA axes are shown; each plot displays a different combination of axes: (a) axes 1 and 2, (b) axes 1 and 3, (c) axes 2 and 3. Approximate area coverage of the four groupings identified by CA (Fig. 5) are similarly color-coded.

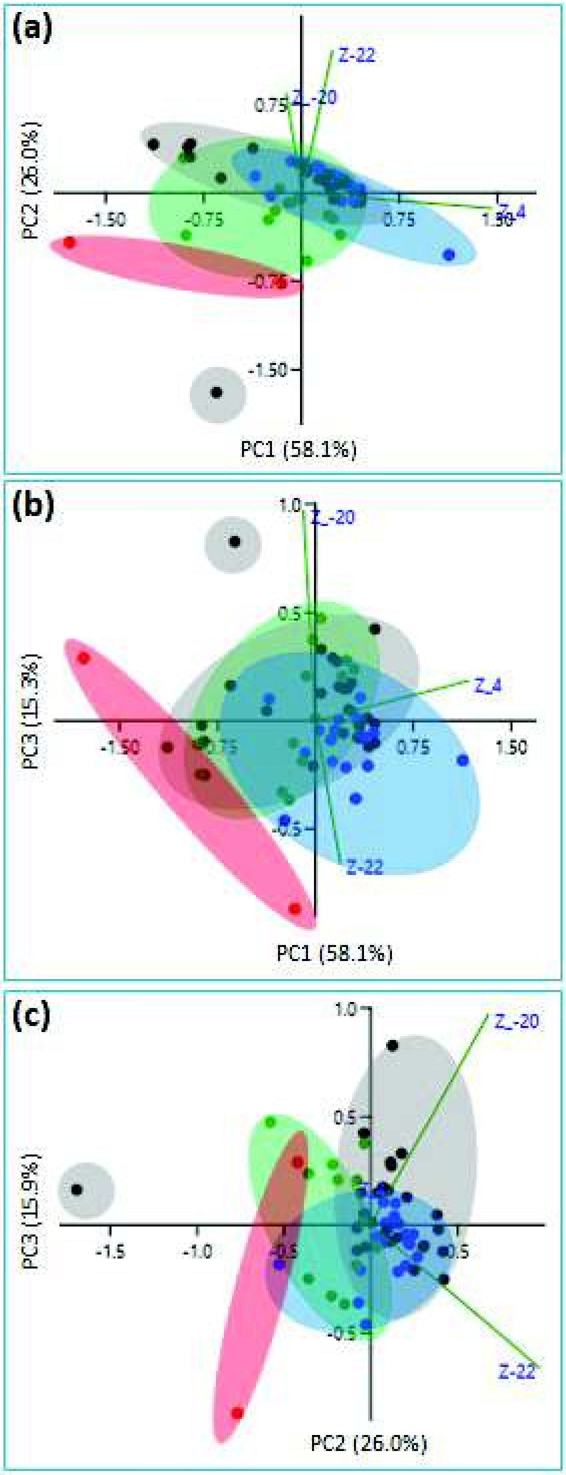

Supplement: Supplementary file 1 — Supplementary Information. [file 41598_2020_74688_MOESM1_ESM.pdf]
